# Genomic data integration for ecological and evolutionary traits in non-model organisms

**DOI:** 10.1186/1471-2164-15-490

**Published:** 2014-07-17

**Authors:** Denis Tagu, John K Colbourne, Nicolas Nègre

**Affiliations:** INRA Rennes, UMR 1349 IGEPP, BP 35327, 35657 Le Rheu Cedex, France; School of Bioscience, University of Birmingham, Birmingham, West Midlands England; Université Montpellier 2, UMR1333 DGIMI, F-34095 Montpellier, France; INRA, UMR1333 DGIMI, F-34095 Montpellier, France

## Abstract

Why is it needed to develop system biology initiatives such as ENCODE on non-model organisms?

## The next generation genomics era includes non-model organisms

Genetics, and now genomics, applied to model organisms continues to be hugely successful at identifying and characterizing DNA elements and mechanisms involved in major biological processes, such as the regulation of development, cell cycle and cell signaling. However, the number of organisms that are supported by large research communities applying genetic approaches is limited. Organisms such as *Escherichia coli*, *Saccharomyces cerevisiae*, *Arabidopsis thaliana*, *Caenorhabditis elegans*, *Drosophila melanogaster, Danio rerio* or *Mus musculus* are elected as “super model organisms” mainly based on their important yet curious biological attributes and many technical advantages. *Drosophila* emerged as the premier study system for genetics because of naturally occurring visible mutants, which led to the discovery of chromosomal heredity, while *Caenorhabditis* was selected as the main organism for studies of cell differentiation and development because its cell lineage is nearly invariant from egg to adult. All are ideal targets for genetics as they are easily reared or cultivated in the lab in order to systematically generate the necessary mutants or genetic crosses. Model organisms in genetics share common traits including short life cycles and a high fertility rate. They are robust cosmopolitan resources of laboratory experiments.

The trade-off to using model organisms is that they are often not “typical” and do not reflect the biology of their close relatives or even the wide diversity of living mechanisms. They also display only a fraction of the traits found in the biosphere, often limited to observations made in the laboratory. Yeast, for example, does not form multicellular hyphae and *A. thaliana* has no known root symbioses. *C. elegans* and *D. melanogaster* are not pathogens or pests and the zebra fish is certainly not adapted to living in marine environments. Similarly, rats and mice, typically used as models in biomedical research, are nocturnal not diurnal. Even commonly used human cell lines, such as HeLa cells show strong rDNA rearrangements [1, 2]. It is perhaps no surprise that over 50% of many genomes of model species are still without experimentally determined functional annotations when many traits are conditionally expressed in varying and natural environments. In addition to the lack of phenotypic representation in model organisms, it is worth noting that many species that either participate in anchoring ecosystems (e.g. keystone species) or are responsible for many health, agronomical and environmental challenges (e.g. human and animal disease causing agents, plant pests, invasive species) are not model organisms.

**Figure 1:**
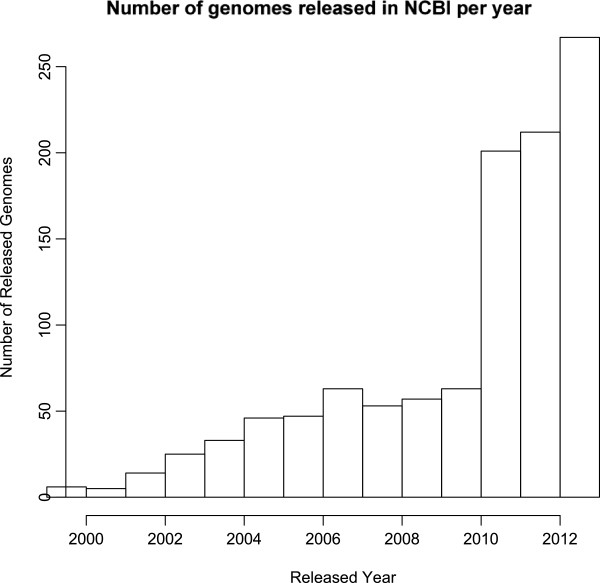
**Histogram of released genome sequences in NCBI per year.** A steady increase can be observed after 2003, with a brutal acceleration after 2010 (data from “ftp://ftp.ncbi.nlm.nih.gov/genomes/GENOME_REPORTS/” downloaded November 2013).

Fortunately, the recent advent of Next Generation Sequencing (NGS) coupled with other high-throughput and high-definition analyses of the cellular organic molecules (compound screening, mass spectrometry) provide the opportunity to rapidly generate genomic, transcriptomic, proteomic and metabolomic resources for potentially any organism and their populations. More than 1,300 eukaryotic genome sequences are archived in NCBI as of April 2014 (ftp://ftp.ncbi.nlm.nih.gov/genomes/GENOME_REPORTS/). Clearly, the number of genome data submissions has steadily risen over recent years, notably between 2009 and 2010 (Figure 1). This trend will persist and accelerate as the cost of sequencing continues to plummet. However, many genome sequencing projects concern species that are closely related to already well-characterized model organisms; of 2,401 listed project at NCBI, only 991 different species from 653 genera are represented thereby illustrating a focus on sequencing genomes from related strains or populations of model species, which benefit from mature genome structure annotations and a wealth of other functional genetic information generated by closely-related model organisms. Nonetheless, research groups studying alternative species, (which are evolutionarily distant from traditional models and usually not amenable to forward genetics), can contribute much new knowledge and important discoveries in this “omics” era, by exploring biodiversity at the molecular level and by describing the natural history of genomes. Several consortia are organized to sequence large swaths of the tree of life (e.g. 10,000 thousands vertebrate genomes, 5,000 arthropod genomes) [3, 4], promising a greater diversity of sequenced genomes within the coming years.

However, this fresh stream of data is too often harvested *ad minima*. Genome sequences are frequently produced solely to obtain gene annotations that are borrowed from functional ontologies for genes in model species. Such borrowed annotations are generally achieved *via* combining computational gene prediction (e.g. Open Reading Frame (ORF) predictions) with sequence alignments and homology comparisons to these well-annotated model organism genomes, including that of humans. Too little investment is presently given to experimentally validate the evolutionary functional conservation of these homologues or to investigate the diversity of genes that are lineage-specific and whose functions are best described in the context of the ecology and evolutionary history of the sequenced organism. Yet just as functional genome annotations in model species are made reliable by comprehensive and systematic investigations, a similar community-level pursuit of a comprehensive and structured comparative genomics knowledge-base is required to understand the biodiversity of genomes.

## Non-model organism genomes beyond genes

The main goal of this paper is to collect thoughts and discussions to initiate community-based genomic data integrations for non-model organisms. We especially wish to discuss (i) how to share genomic knowledge obtained via different technologies, and provide useful comparisons metrics for scientific discoveries linking genome structures to biological functions, (ii) guidelines for the different phases or steps of these projects (see below), and (iii) types of data needed for additional annotations of genomes that may reach beyond individual labs to generate otherwise difficult comparative and important biological findings, derived from a collective effort.

The rationale for these thoughts is based on the recent history of genome sequencing projects and the fact that functional annotation of DNA elements, especially targets of natural selection, is needed for non-model organisms. It is becoming clear that valuable gene-by-gene approaches to molecular biology under uniform environmental conditions are not ideal when the scientific goal is to elucidate whole biochemical pathways, or emergent features of cellular and organismal biology that are expressed and have evolved under varying environmental conditions. Molecules present in cells, tissues and organs function within integrated systems [5]. It is the multiplicity of regulatory interactions within and between units – whether among cells or individuals responding to environmental conditions – that gives rise to complexities of biological organizations. The genetic component of the phenotypes of interest depends on variations across many domains beyond the coding regions of the genome. Regulatory elements, chromosome and chromatin architecture, repeat sequences mobilization, all have roles to play in building morphological and behavioral traits. In most assemblies for which we have a high quality annotation, exons represent only a fraction of the genome (in *Drosophila*, 25.7% of the 169 Mb of sequenced genome is comprised of exons; compare to 1.5% in the Human genome). This huge amount of unannotated sequence data potentially harbors key elements of the genetic underpinnings of phenotypes, if only their full functional repertoires were known. The ENCODE (ENCyclopedia Of DNA Elements) research programme, led by the National Human Genome Research Institute, explored the definition of “gene” in the light of functional genomics and systems biology data sets [6, 7]. The notional definitions of “gene” reasonably vary between disciplines, and evolve with technological and methodological innovations. Traditional genetic approaches identify gene products that are necessary and sufficient for an expressed phenotype. Yet in terms of “function”, the definition of gene transcends its capacity to produce a transcript, or a protein. Additional knowledge of genomic variation segregating within and among populations with known ecologies, of regulatory elements and the networks that govern gene expressions under both neutral and adaptive evolution reveals the importance of alternative signatures to the functional attributes of DNA elements. Genome plasticity observed in animals and plants is likely to reveal new functional elements that are necessary for the expression of fitness related traits. Indeed this path to discovery may be amplified when studying the genomes of populations and the organismal molecular responses to their environmental challenges.

In the early 2000s, communities working on model organisms anticipated the tremendous power of nucleic acid sequencing and protein characterization; these communities aimed to catalog all the interactions through which DNA elements influence cellular and organismal biology. ENCODE [6, 7] is one consortium-led initiative that was launched soon after the publications of the human genome, in order to identify (annotate) the entire DNA landscape of functional elements in this genome. This was (still is) a progressive stage that follows other post-genome sequencing initiatives such as the HapMap consortium project describing human genome diversity [8] - further supplemented by the 1,000 genomes Project [9] - or the Human Microbiome Project [10] that compiles microbe interactions in either healthy or diseased humans. Of course there are recent phylogenomics initiatives too describing the evolutionary history of the human genome by comparing it to other primates, mammalians or other species genomes. ENCODE is a top-down initiative, with open calls for proposals by biomedical research groups that conceived of novel approaches to reach the project’s goals. ModENCODE was developed as pilot experiments using *C. elegans* [11] and *D. melanogaster* [12] to set up tools and to check for the feasibility of the ENCODE programme, much as the shot-gun *D. melanogaster* genome project [13] was a pilot experiment for the shot-gun human genome [14, 15]. Now that the ENCODE initiative enters into its second phase, integrations between HapMap, HMP and ENCODE should give positive and added value to post-genome sequencing projects, a kind of 3.0 step of genomics (1.0 being the genome project, 2.0 the individual ENCODE, HapMap, HMP projects).

For these reasons, ecology, evolutionary biology or diversity in its outputs were not considered parts of the modENCODE and ENCODE projects, which were mostly based on developmental and cell biology as a basis for addressing biomedical issues. Most annotations of the functional DNA elements revealed by ENCODE follows an ontogenic vocabulary adapted to biomedical research, although the notion of functional DNA in ENCODE has been long debated [16, 17]. However this so-called ‘Gene Ontology’ (GO) does not completely encapsulate ecological traits for describing biological processes, cell components and molecular functions. This is a critical discrepancy of the GO since finally, even for model organisms with well-known genetics techniques and many mutants, the function of many predicted genes is still unknown. For example, 7,574 *D. melanogaster* genes out of its total set of 16,656 genes (Release 5.54) are still “Computed Genes” with no observed phenotypes. Initial genomics investigations of orthologous genes in ecological model species are pointing to many “Computed Genes” being expressed under narrowly defined ecological conditions that are not experienced in laboratory settings. This observation has consequences for the annotation of sequenced genomes of non-model organisms, since their annotation is mainly based on orthology with genes from model organisms. For example, in the sequencing project of the Monarch Butterfly (*Danaus plexippus*), a set of 16,866 predicted genes is presented [18]. Of these, roughly 1,000 gene annotations are manually scrutinized. Those validated genes are chosen based on *a priori* expectations that they have central roles in the butterfly’s migration. The authors supplemented their analysis with one type of regulatory mechanism (microRNA), hinting that there are more discoveries to be made. Such a fascinating investigation would greatly benefit from resources to facilitate a more complete annotation of genes and regulatory elements linked to migration. And in turn, a knowledge-base that included annotations made in light of the different ecological conditions and behaviors experienced by non-model organisms (such as migration) will help improve gene annotation of other non-model and model species alike.

## The case for systems biology initiatives on non-model organisms

Large consortia projects often face many criticisms [19, 20]. For example, although ENCODE consortia were able to deliver several useful achievements, perhaps the focus should have been to promote the resource as much as the findings. The integration of different assays from molecular biology conducted with uniform protocols on a controlled set of samples made it possible to develop several tiers of genetic and epigenetic information in the same systems. Similar utility has been achieved by the RoadMap Epigenome Project, the BluePrint Epigenome Project, and others. Many DNA elements of various types have been mapped and annotated, with the objective to establish interactions [12]. Already, discoveries are made from this data integration of the experimentally derived functional annotations provided by ENCODE and modENCODE encode projects. In *Drosophila* modENCODE for example, a class of DNA elements termed HOT regions for High Occupancy Targets is better understood [21]. These elements were described by modENCODE as regions in the genome where a large number of different Transcription Factors (TF) co-localize [12, 22]. Thanks to the sequence motif signature of these HOT regions, further work has independently demonstrated that they are regulated by a single TF – crucial for the maternal to zygotic transcription – and that HOT regions define a specific class of functional enhancers [23].

ENCODE-like annotations are now a valuable integrated resource used by many to advance their research in model organisms. We argue that the next step is to understand how the genetic system reacts in its full natural context: when the developing organism is subject to all the challenges of, and interactions with, its ecosystem. Thus, it becomes important to promote data-driven modeling of all these environmentally conditioned assays through ENCODE-like initiatives for non-model-organisms, having knowledge of their ecology. We refer to this approach as neoENCODE: the functional annotation of genomes applied to emerging and non-model organisms, which are typically not amenable to large-scale forward genetics. And, just as the “one gene” approach is insufficient to investigate genome-wide transcriptional control, the “one organism” approach also has its limitations. Sequencing and imaging technologies can be used to identify key players in the establishment of phenotypic traits that govern inter-species relationships and give rise to local and global ecologies. We propose that investigations in these and related areas are well-positioned to transform our understanding of both organismal and population biology by providing much-needed context.

**Figure 2:**
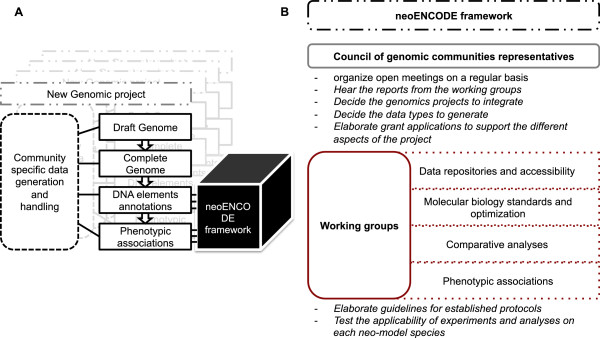
**A proposal of neoENCODE organization. A**. The neoENCODE framework will not take part in electing neo-model species nor in organizing genome-sequencing consortia. It will act at the level of annotation and phenotypic association as a common integrative framework for many genome projects. **B**. In order to give the neoENCODE framework more substance, a council of representatives from the different communities involved could chair this initiative. They could organize regular open meetings and hear the reports from the other structuring component of this framework: the working groups. Each of this group could work on experimental and analytical aspects of the project. Some areas where they could be involved are highlighted on this figure. Their main task will be to create common guidelines for established procedures, but also, ensure that those procedures are applicable to the peculiarities of each neo-model species.

To clarify, the proposed neoENCODE (Figure 2) has a different structure and possibly a different mission to modENCODE and ENCODE: neoENCODE is proposed to be a bottom-up strategy (see below) because the communities involved define their own needs and requirements, which are adapted to the size of the community and biological specificities of the species of interest. Funding support for these diverse neoENCODE initiatives will also differ for each community. Yet the common thread that links the activities of these communities into a neoENCODE research programme is a shared road-map and a shared knowledge-base infrastructure for integrating the pooled data from participating communities (see below). We can anticipate that some communities are sufficiently well mobilized to begin this data integration sooner than others, for example, those studying a first wave of ecological and evolutionary genomics neo-model species, such as the honey bee, *Daphnia*, aphid or the sea-urchin to name only a few. neoENCODEs have the flexibility to be structured at different levels: to accomplish large and ambitious projects, planned across the timescale of a decade, or to realize smaller and targeted projects addressing specific questions. This federated and flexible approach at filling the current knowledge gap on the diversity and function of genome structures in light of ecology and evolution benefits from lessons learned from over 10 years of community-coordinated genomics research, which values bold objectives and relies on interdisciplinary teamwork to accomplish them. A distinguishing feature of neoENCODE is how, with the right strategic investments that expand existing database and information sharing infrastructure, its organization is permitted to evolve with the number neoENCODEs and the scale of their projects. As described below, we need road maps and exchanges between neoENCODEs communities to improve each of the individual neoENCODE projects.

Another distinguishing feature of neoENCODE is the point at which a project is completed, or the results deemed to be comprehensive. ENCODE and modENCODE had/have the ambition to screen a number of organs, tissues, cells and biotic and abiotic challenges, mainly in laboratory conditions, for empowering biomedical research. By contrast, NeoENCODE will endeavor to largely replicate many *ecological conditions* across a wide array of non-model species either under controlled conditions or directly from natural populations. Therefore neoENCODE will strengthen environmental research knowledge on the environmental associations with gene functions and phenotypes, including disease.

In short, we propose herein a discussion on the application of such an integrative approach using functional genomics and post-genome sequencing data from non-model organisms. This refers to ENCODE and modENCODE initiatives but with a different perspective. Our proposed initiative is not to create a new consortium, structured and piloted by a coordinating group: this approach applies more to several bottom-up initiatives that several communities might adopt, adapt and organize as they wish, independently from one to another. With moderate size of consortium – that self organizes - this should limit the management difficulties and favor positive collaborative actions. However, meetings (such as workshops, summer-schools) between those different focused initiatives could be efficient to share expertise and best practices. Effectively, we thus do not propose any cues for putative funding or sharing efforts (such as on bioinformatics) since each community could organize it depending on its own objectives and contexts. But we defend the idea that independent bottom-up neoENCODE initiatives requires sharing common goals, practices and knowledge. Road maps are thus necessary to the set up and are described below.

## Technical considerations for neoENCODE initiatives

The second part of this paper proposes (but does not impose) road maps to help communities to consider the added values of genomic data integration developed on non-model organisms. If ENCODE is a map of data resources (as suggested by Eddie [17] and we agree with this suggestion), then neoENCODE will also consist of maps, as global descriptions of elements positioned on the genome that might interact in a cell. We propose a common view for integration of genomic data at several levels that can also be used to design new projects. These different integration levels could concern expressed DNA elements, as well as tissue organization, time-scaled and developmental processes, as well as inter-individual comparisons and inter-species comparisons, given the high ecological and evolutionary knowledge of the species of interest.

Technically, working with non-model organisms means there is limited access to high-throughput functional assays. Thus, the most effective approach for deciphering genomics mechanisms from non-model organisms is to integrate comparative data providing annotations of DNA, RNA, and protein features. From a reference genome, thanks to the work of several consortia, a clearer picture of the gene structures, numbers, regulation and gene orthologs emerges that are expected to be found across phylogenetic distances from congenerics to organism representing different phyla. It is easy to anticipate that soon, the mapping of chromatin states, RNA, protein and metabolite content (epigenetics, transcriptional, post-transcriptional…) will be possible for a wide variety of tissues and conditions for many, or even most organisms. We argue that the most important contribution of consortium scale biology, particularly of the ENCODE Consortium, has been the establishment of uniform data quality standards and assay conditions, which have rendered thousands of experiments directly comparable in several systems. We thus have the opportunity here to rally such projects through a proposition of guidelines for data generation and data exchange. This again can be done utilizing some of the ENCODE consortia principles [24].

Before going into a more detailed presentation of a putative road-map for neoENCODE, we propose to keep in mind four technical differences between a neoENCODE initiative and the now-concluded modENCODE projects:

- For most non-model organisms, the size of the scientific community will be much smaller than the communities working on model systems or human. This means that it will be difficult to be as comprehensive as modENCODE or ENCODE; we can predict that the efficiency of a neoENCODE initiative will be strengthened when the dedicated community focuses on specific traits that each non-model species will make particularly accessible and amenable for study.
- It is probable that no cell lines will be available for most of the non-model species. This will complicate the interpretation of data sets. However, it can actually provide added value if key tissues, organ systems and conditions are targeted at the beginning of the project to, again, focus on specific biological processes.
- As discussed above, high throughput functional assays are not systematically available for non-model organisms. The knowledge provided by a neoENCODE initiative will provide a roadmap for these, and will generate the toolset needed for other studies in ecological genomics. This points toward an economic argument for funding such a project at this time, during the emergence of this exciting new field [25] we have the opportunity to establish publically available biochemical and analytical toolsets that will be useful to many researches around the world.
- One of the main added values to genome studies is the access to natural populations, that is often covered by population geneticists. Including DNA polymorphism diversity within the neoENCODE projects can strengthen both the upstream steps (including individual variation as a sample to produce sequencing data) and downstream steps (using natural variants as a source of functional assays/validation) (see for technique: [26]). This is what is occurring for *Homo sapiens* as ENCODE data is integrated with the HapMap [27] and other population-genetics resources.

## A roadmap proposal for neoENCODE project

We think it is time to rally the different communities and call to genomicists, biologists and computational scientists. All those who are interested in understanding how genomes interface with the natural world need to join forces on a neoENCODE project. Genomic projects in model organisms are well advanced and we can refer to 4 main phases that described this genome projects (Figure 2A):

Phase 1: draft genome (consortia for *C. elegans*, *D. melanogaster*, *H. sapiens*, *M musculus*) that involved the choice of models as technical roadmaps of feasibility.

Phase 2: finished genome assemblies and annotations,

Phase 3: annotation of DNA elements (pilot ENCODE, modENCODE, ENCODE, mouseENCODE),

Phase 4: phenotypic annotation of the genomes especially in Human with the use of population data for GWAS studies, focus on medical trait.

The road-map we propose for neoENCODE integrates this historical knowledge and tries to adapt it to the intricacies of these future projects (see above). However, given the million of species present on earth, how can neoENCODE strategies be realistic and scale? Below are listed a series of steps we think will be crucial to consider:

### Learn from and integrate existing, planned and future genome sequencing projects

Large genome project initiatives such as 1001 Arabidopsis genomes (http://www.1001genomes.org), 1000 fungal genomes (http://1000.fungalgenomes.org/home/), 10000 vertebrate genomes (https://genome10k.soe.ucsc.edu/) or 5000 arthropod genomes (http://www.arthropodgenomes.org/wiki/i5K), to list only a few, are probably the melting pots to incubate the future neoENCODE programmes. Discussions in these communities are already tackling issues such as harmonization of protocols and standards. This harmonization should probably be done through common portals and interfaces allowing for future common annotations-types and formats. A genome is never completely assembled or annotated. But assembly and annotation efforts need to be maintained for genomes entering the stage of functional annotations. This foundational step establishing uniform genome quality is already clearly needed, and much has been done to bring it about [28].

### Prioritize the systems

Obviously it is the duty of each concerned community to elect the organisms they want to explore with functional genomics technologies. The reasons beyond the choice of model vary from case to case: mainly economical (agriculture, medicine, conservation…), or of evolutionary importance (the first non-primate mammal, the first cetacean, etc.…).

It will be essential for researchers working in new systems to ascertain good material production pipelines, with the capacity to perform multiple biological replicates, as well as organ, tissue, and cell-type dissections and mass isolations (see below).

### Choose the experimental conditions and targeted organs and tissues

One of the aims of neoENCODE strategy is to focus on defined, novel and ecological traits, not to re-analyze traits that have already been efficiently characterized in model organisms. The choice of particular targets for organ or tissues dissections is thus essential for i) originality, ii) designing focused experiments, iii) limiting costs, iv) eventually limiting the complexity of the targets in terms of cell type diversity that might complicate expression data interpretation. Choosing the right organisms and targets is essential also to reduce the risk of redundancy between species. Cell lines do have technical advantages (relative homogeneity, large quantity, more amenable to RNAi, etc.) but also one significant disadvantage: they do not fully reproduce the phenotypes of the tissues from which they are derived [29]. That is why, in the scope of a neoENCODE project, a particular emphasis should be placed on the choice of target organ systems and temporal windows for each species. While keeping the uniqueness of each species in mind, we also need to choose tissues and/or developmental time-points that will allow direct and interpretable comparisons between organisms.

### Choose the common data-types to produce

One goal here is to cover many different steps in the hierarchy of gene function and regulation. The first, basal layer of data to produce is of course transcriptomics, including coding and non-coding RNAs (mRNAs, rRNAs, tRNAs, snoRNAs, miRNAs, siRNAs, piRNAs and equivalent, long non-coding RNAs).

Beyond RNA content are the mechanisms of gene regulation. Another aim of the ENCODE projects was the annotation of regulatory elements. It has been shown that general chromatin information captured by assays such as FAIRE-, MAINE- and DNase-Seq [30, 31] is indicative of gene regulation function, and these assays have been used to identify active promoters and enhancers. Such experiments can be performed in priority and easily on most new-models. Still, these experiments require a larger amount of biological material (up to 10^8^ cells in the case of DNase-seq) and more protocol optimization than for simpler RNA extraction. A third type of experiment is of course ChIP-seq, used to reveal regions bound by regulatory or structural proteins. Of these we distinguish Histones (and more particularly their post-translational modifications) and Transcription Factors (TFs). The difficulty is that there are hundreds of histone modifications, with many new described all the time (e.g. [32]). Animal genomes annotated so far encode between one and three thousand transcription factors, and new species, particularly those with large genomes, may encode many more. This figure is currently out of reach for a neoENCODE project. Pioneering work performed on *Drosophila melanogaster* shows that chromatin marks can be grouped into five different biological states [33]. Later, modENCODE provided a 9-state and even a 30-state model [12, 34] representative of different classes of DNA Elements. Across organisms, the Histone modification H3K4me3 seems generally to be a good marker for active transcription start sites. Similarly, CTCF, in Mammals and Drosophilids, seems to be the main insulator protein, even though exceptions occur [35, 36]. *C. elegans*, for example doesn’t possess CTCF [37]. With this in mind, we propose to select a small set of conserved TFs and Histone modifications to perform systematic ChIP analyses in all the selected new models in order to annotate candidate regions linked to gene repression or activation. One key difficulty will be the availability of antibodies for new organisms. Working with established industrial collaborators, will be essential to enable high-throughput functional genomics in new systems.

These studies can of course be complemented by analyzing post-transcriptional regulation by RNA binding proteins (RBPs). There are between half (humans [38]) and two third (flies [39]) as many RBPs as TFs in most annotated animal genomes. And of course, there are many other forms of regulation as well (pre-post transcription…), e.g. DNA methylation, chromosomes conformation and structural regulation (the role of centromeres and telomeres for example), or even transposable elements hopping. Other steps of gene regulation can be investigated by polysome extraction and RNA sequencing to determine the pool of RNAs that are under translation (associated with polysomes), but this has proved difficult to transfer between species, due to the need for extensive optimization [40].

### Build the computational pipelines

**Bioinformatics begins with a genome database**: gene expression data is best analyzed in reference to a genome annotation. This means that a dedicated database is required. Usually, raw data are cleaned and mapped to a reference genome. These steps are now pretty well-defined and do not cause difficulties for trained scientists. In all cases, the multiplicity of samples requires access to substantial computational and storage capacities. Bioinformatic platforms and/or data centers are necessary for accomplishing these analyses. Of course, the neoENCODE project needs the involvement of trained computer scientists, and trained genomicists in analysts and programmers. Systems such as Galaxy [28] allow biologists, after brief training, to handle large quantities of data and perform some analyses. Hence, there exist excellent models for what we aim to accomplish. Again, herein we do not pretend to propose a unified bioinformatic platform: we think each neoENCODE community will organize their computational infrastructure before joining the project.

**But the final critical** step of a neoENCODE strategy will be software development allowing data integration and comparison through a wide range of species. Mathematical and statistical modeling is probably not the main skill of most researchers in molecular biology and ecology, and neither is it for the authors of this tribune. Thus, collaboration with modelers is necessary, and, hence, it will be important to work with individuals with mixed biological, mathematical, and/or computational training, who can serve as conduits between the white-board, the computing cluster, and the wet-bench. This will require a dialog between different experts. And this step can be also more feasible by beginning integration of a small number of objects (2 or 3 such as mRNA, miRNA, TF) and to step by step enrich the models adding new data (DNA methylation…).

## Conclusions

Many choices will need to be made in order to initiate neoENCODE initiatives at an international scale on the ecological genomics new era. One of them is the interest of industries in such initiatives. The direct consequences of such neoENCODE or genomics data integration for industry and economical activities are still difficult to evaluate, but at least this can be included in most of the societal challenges defined for instance in Europe by the European Community: “Health, Demographic Change and Wellbeing” for biomedical research, “Food security, sustainable agriculture and forestry, marine and maritime and inland water research and the bioeconomy” for agronomical research, “Climate Action, Resource Efficiency and Raw Materials” for environmental research are examples. These societal challenges reflect the general mid- and long-terms goals our societies need to reach, and probably followed by economical actors and stake holders. Whether such neoENCODE strategies will be i) useful for private companies and/or ii) used in their R & D programmes will be rapidly evaluated when funding will be tracked by academic groups.

If we are to begin to dissect the interface between the biology of cells, organisms, and environments, we suggest that a federated collaboration model will yield a cost-effective path forward. One limitation of the strategy we propose will be the size of the scientific community with expertise in individual organismal and ecological systems. Hence, out-reach will be critical to our success, and we hope that this tribune is the beginning of a vigorous dialog that may culminate in the generation of several neoENCODE white papers dedicated on given organisms and biological questions. If we begin early to rally our forces, we may have the opportunity to kick off the era of ecological genomics the right way: as a unified, cost-effective community with efficient communication and global data-quality and analysis standards. In the very next future, one can expect that discussion on this tribune will fuel the progress of neoENCODE strategies, and we encourage readers to organize local or international workshops to explore these new possibilities and disseminate them in our community of structural, functional, evolutional and ecological genomicists. As well, common journals or group of journals (such as BioMed Central) might also be used to strengthen to connections between neoENCODE initiatives.

## Reviewers’ comments

We thank the reviewers for their comments on an earlier version of this Correspondence article.

### Reviewer 1 (name withheld)

This opinion article is clearly intended to initiate a conversation within the biological community regarding the contribution of advances in genomic science to our understanding of biological systems beyond traditional model organisms.

The authors first emphasize the value of the ENCODE and mod-ENCODE projects which have assembled an impressive catalogue of genomic features, molecular interactions, etc., outlining the critical service these projects have provided to the human and model organism community. They also argue that the handful of traditional model organisms currently available only allow us to address a narrow set of biological problems. They raise the question of whether or not Encode-like projects should be democratized to a wider range of so-called non-model organisms arguing for a resounding “yes”. Their argument centers on the idea that our recent understanding of bio-complexity has changed how we should study biological problems. The very knowledge that was gained through the (mod)-ENCODE projects and the complexity we have uncovered should motivate similar studies following the same framework that would allow the integration of data across various levels of biological organization.

Unfortunately, I am not convinced the authors have clearly articulated what the goals and outcome of various non-mod-ENCODE initiatives would be, especially given the limitations outlined at the end of the article. I realize that space is limited, and that this is not a white paper, but outlining even briefly (with an example maybe) how they envision such a project would materialize (not financially but practically) would be tremendously helpful in convincing the reader. If my organism of interest were for example, a scale insect, what would a non-mod-ENCODE initiative look like? What data would be collected, how and what kind of biological questions such a complex catalogue would serve to address. How many non-model organisms should this be done for? How should they be selected? Since there are so many potential non-model organisms, these are relevant and complicated questions that should be addressed, especially when the relative value of the effort is very difficult to appreciate ahead of time. It would seem given the limited resources we have to work with that we would be adding a few more organisms to the list of model organisms rather than really expanding into (many) non-model organisms. I suppose if resources were unlimited then, non-modENCODE (I’d prefer beyond-modENCODE) would be great, but resources are never unlimited. And since high throughput next-gen tools are available to any lab, scientists studying non-model organisms have plenty of tools at their disposal to start to study adaptive evolution (for example) without a huge community effort like (non)modENCODE.

My concern however is with this statement that seems to serve as the key rational for such projects: “Technically, working with non-model organisms means there is no access to high throughput functional assays. Thus, the most effective approach for deciphering the complex adaptive biological mechanisms of non-model organism is an ENCODE-like strategy”. First, I should emphasize that it is still unclear what the mod-ENCODE projects have accomplished toward this goal in model organisms (and if it is clear to the author I urge them to explain how). We still have a very long way to go to interpret mod-ENCODE data in that context. Most of the data collected to date, for example, do not even consider the contribution of genetic variation or genotype-by-environmental interactions, which could not be ignored for non-model organisms. It would appear that what the authors are arguing for is not a non-mod-ENCODE project (and all that it entails – need for controlled environmental conditions, assay of developmental stages, in different tissues, etc.…). Instead they advocate the development of genomic resources that would enable the application of system-biology and system-genetics to investigate complex biological problems that are specific to certain groups of organisms. I do understand the appeal of following in the footsteps of a successful initiative, but this paper would be much improved if realistic and more specific goals were outlined clearly. The answer to the questions posed in the introduction [“what is the point of a catalog of molecules without demonstration of their functional roles? Is it worth getting such an amount of purely descriptive data from non-model organisms without having access to functional assays such as forward genetics or transgenesis? Is it worth investing in the accumulation of terabytes of data from non-model organisms?”] can be answered with a simple “yes” because biology is complex and genes do not act in isolation but as genetic networks.

Advances in genomic technologies have without a doubt changed many fields in biology, and the holy grail of understanding the genotype to phenotype map in functional terms within the context of the organism has never seemed so close. As stated in this article, there is much to be gained by bringing together disciplines that have traditionally been isolated. Many of these new tools that are not available have given us a false sense of confidence with respect to what we actually understand. This trend probably started with the popularization of microarrays, when a list of differentially expressed genes became synonymous with genetic architecture and to many redefined causality in biological experiments. I think if the authors are to convince a large community that considerable resources should be dedicated to cataloguing in great detail the genome of non-model organisms (an endeavor I would personally gladly support), the grand vision outlined in this paper would be much better served with a careful justification with specific and practical goals.

### Reviewer 2

Mark Blaxter

School of Biological Sciences, The University of Edinburgh

This is an interesting opinion piece that suggests ‘upscaling’ our genomics approaches on non-model organisms, and building ENCODE-like programmes to deliver ‘systems biology’ level understanding of a wider range of organisms than those currently studied.

The point of models is that they are taken to represent the core biology of all species in a particular realm: so we can have model species that model humans, or that model development in a particular group, or that model genetic processes.

It is perhaps necessary to revisit the model when a phenotype of interest is not represented in the core set, but analysis of these phenotypes can be achieved in new or satellite models that do have the relevant biology.

The thesis is intriguing, but is poorly focussed, and perhaps oversells the knowledge that can be mined from the ENCODE data systems. I am not impressed by the bite or reach of the arguments made, and the simplistic call for ENCODE-like programmes on other organisms could conflict with exploitation of the true utility of these as systems in which population genetics can deliver real answers to questions of pattern and process in the natural world.

Some comments:

- What has ENCODE actually done?

ENCODE has analysed the genome wide distribution of epigenetic marks, chromatin states and of transcription factor binding sites in humans, *D. melanogaster* and *C. elegans*. Similar programmes are effectively underway for key bacterial and fungal species (but here the ease of culture, single-celled target and size of the genome means that the programmes are not vast in extent). Yes, the majority of the human genome was ‘tagged’ by one or many different analyses, but the claim that this means a “biochemical function” for the majority of the genome has been defined is a little overblown. In a large proportion of cases the mark that has been identified is one that results in silencing and transcriptional inactivity: so the “function” is to be devoid of positive function. Secondly, the ENCODE analyses are built on deep layers of previous functional genomics work, and are in essence a series of correlations: the following epigenetic marks correlate with such and such other features of the genome. By performing these analyses across many cell lines of different phenotypes the ENCODE teams were able to infer the logical structures of many of the core regulatory systems in the human, and to identify where likely disease-causing variations were likely to occur, and provide frameworks on which to build models of disease (and health) causation.

In doing these analyses ENCODE has built new suites of analytic tools and approaches to huge data that are likely to be of general applicability.

(b) what are the target non-model organisms?

It is unclear what the non-model organism set being proposed is. There are ~10 million animal species on earth; 1 million have Linnaean names. There are, in addition many, many million more plants, fungi, various phyla of protozoa/protoctista, bacteria, archaea and the soup that is the viral world. Of these millions, perhaps 10,000 are seriously studied at the genomic level. Many of these studied species are already proposed as satellite or new model organisms, specifically to model pieces of phenotype space that the core models cannot. It is very likely that encode-like programmes could be carried out on this much-restricted non-core model set, as long as the need to understand the system in detail meshes with the accessibility of the organism/phenotypes being studied.

(c) is an ENCODE approach technically possible?

Given good reference genome sequence data, many of the core analyses performed in ENCODE could be repeated on any organism - looking for DNA methylation marks using custom antibodies or chemical modification to separate the Me marks from the unmarked DNA, looking for open chromatin using DNAse1, or fully cross-reactive anti-modified histone antibodies to identify histone marks. However much of the specific data generated in ENCODE used pecific immunoprecipitation using antibodies generated against a series of transcription factors. These reagents are not simply transferrable across species (and indeed much of the work leading up to ENCODE was proving the exquisite specificity of these reagents). Developing these anew is not trivial.

ENCODE (and, to some extent, Drosophila modENCODE, but not *C. Elegans* modENCODE) relied on cell lines - clonal growths of isolates with a stable phenotype. Cell lines are not available for most taxa (there are none for *C. elegans*, for example) and generating cell lines is not trivial (*C. elegans* is a case in point -the lack of cell lines is not for want of trying). modENCODE relied much more on tissue and whole organism samples, and this is likely to be true of any “nonmod” programmes also. This inability to move to the cellular level will limit the systems biology that can be done.

That said, many “new-model” organisms are effectively being “encoded” – the honeybee, many farm animals and agricultural crops, some satellite models of the key species… all have extensive programmes of systems research underway.

(d) an elephant in the room

I was surprised at the emphasis on adaptive evolution in the text. It is clear that the shape of any genome, and the phenotype of any organism, is the outcome of both selection and neutral processes (including hitchhiking, bottlenecks, etc.). To suggest that the structure of the systems biology models that emerge from ENCODE reveal adaptation only is overly Panglossian: much of the structure must arise from stochastic fixation and drift. The null hypothesis in any analytic experiment that is looking at evolutionary change must be that the pattern observed is due to neutral processes.

(e) what are non-model/new-model organism researchers doing?

Looking across the new model organisms, and across the wider field of the use of organisms that are not the core models, several things are evident. The first is that the questions being asked and answered may not NEED genomics or functional genomics data to be accurate and informative. The second is that in many cases the approach taken is that of population genetics: identifying variation of interest within or between species, and then using whole organism genetics to identify the proportion of the variation that is genetic, and then using quantitative trait or association genetics to identify the genetic structure of the trait, and the dynamics of trait evolution. It may be of interest then to go to the genomic level, and to identify quantitative trait nucleotides, and investigate the biological mechanisms (at the levels of transcription, translation, protein function, cell phenotypes, tissue organisation/cell communication and organismal physiology) that underpin the variation observed. On the other hand it may not.

Other notes:

The language of the article needs some cleaning, and there are several sentences where I was unable to work out what was meant.

- “ a DNA element functional in its wider acceptation”
- “description of biodiversity at a molecular level”
- “clearly modified or clarified the notion”

In addition there were several claims of fact or generalisations that were not supported by reference and seem to me to be dubious:

- “mice are not adapted to spontaneous disease”
- “adaptive evolution … often implies the mobilisation of transposable elements”
- “added values in non-models … is access to natural populations” (surely the history of work on humans and Drosophila is significantly driven by natural population work?)

There are some trivial statements:

“most living organisms … are non-model organisms” (by definition, surely)

### Reviewer 3

Roderic Guigó

Centre for Genomic Regulation (CRG), Barcelona

The proposal for a non-mod-ENCODE, that is, for ENCODE like initiatives for non model organisms is very timely. Therefore I fully support the message in the manuscript. On the wave of the recent publication of the ENCODE manuscripts and of the upcoming publication of the comparative ENCODE modENCODE results, the publication of the manuscript is particularly appropriate. I believe that I mostly agree with the main reason for non-mod-ENCODE projects: “We have no choice”. Obviously, I would have written a different paper, emphasizing different topics. Thus, my differences with the manuscript are mostly of style rather than content.

There are a couple of areas, in which the authors could make additional emphasis. Please, take these just as suggestions:

First. Very few people today still hold the position that genome sequencing (of novel species) is a waste of resources. However, to fully mine a genome sequence, we need to have information about its function/activity. Recent advances in Next Generation Sequencing makes affordable overlaying transcriptomic and epigenomic information onto a given genome sequence in a diverse panel of cell types, and conditions.

Second. The authors cite as a limitation in non model organisms, the lack of high throughput functional assays. This is actually a strong motivation for ENCODE like projects. It is not only a cost-effective way to understand genome function on these organisms, it is the only way.

Third. One of the main outcomes of the ENCODE and modENCODE, beyond the biological insights that they have produced, is the delineation of standards and recommended practices, both regarding experimental and computational procedures. In fact, a large part of the ENCODE effort has been likely devoted to this. This has been extremely beneficial for the community, and in particular for any nonmod-ENCODE project. The authors allude to this, when they mention epigenetic markers already used in ENCODE, but they could go beyond this and include many other features of the experimental and computational pipelines (for instance, the use of replicates and of statistical methods to focus analysis on reproducible data, the QC metrics, or the recommended computational protocols for mapping, ChIPSeq and RNASeq analysis. The availability of these standards and recommended protocols is what makes non-mod-ENCODE projects possible.

Fourth. It is impossible to understand a genome in isolation. Life is a continuous. And as powerful as comparative genomics has been to understand the human genome itself, and the genomes of important medical and socio-economic value to humans, comparative transcriptomics and epigenomics—that non-mod-ENCODE projects would make possible—will be to understand the functioning of these genomes of such direct practical importance to us. I think this is also a strong argument for non-mod-ENCODE projects Fifth. The ENCODE and modENCODE projects are largely cooperative projects involving hundreds of scientists working in distant parts of the globe. This is creating a new culture of cooperation and sharing that could be very positive for smaller communities.

A few additional minor comments:

1. page 1.“… provides the opportunity to heavily an even exhaustively describe the availability of different DNA-associated epigenetic and chromatin states”. “describe the availability” sound odd to me
2. The authors bring Biomathematics as providing gene networks allowing biologist to integrate the data. However, simpler models, for instance, predicting gene expression from histone modification data are already very useful.

### Reviewer 4

James Ben Brown

University of California, Berkeley

This correspondence piece attempts to establish and initiate a dialog surrounding a putative new international initiative in ecological genomics. This is a very important and worthwhile thing to attempt.

However, the paper is not yet of publishable quality: the language needs serious work, and there are a number of errors and misconceptions regarding functional genomics. Also, the authors make it sound like the ENCODE and modENCODE consortia have been the only motive force in functional genomics for decades, which is far from true.

Because I am extremely keen on the topic the authors have elected to champion, I have made edits in Word, in track-changes, something I have never before taken the time to do as a reviewer. Please find attached a detailed review, in track-changes, with comments throughout. I hope that this will be of help to the authors, and will aid them in their very, very worthy quest.

### Reviewer 5

Brian Oliver

The National Institutes of Health (NIH)

Proposing a more complete annotation of additional genomes is an interesting idea and well worth a public debate. Collectively, the authors have experience in both “non-genetic” model systems and in high throughput annotation projects and as such are a good team for voicing an opinion.

I think that they could make some points more directly and I have a few suggestions for them to consider, but it is their paper and opinion, so I do not think it is really appropriate to make any of these mandatory. They are just suggestions and observation for them to consider.

1. The significance section is the most important part of the document, so it should probably be several pages long. As it stands, there are three poorly developed concepts that are all good, but all tersely presented and vaguely documented. This makes the first part of the piece quite muddy and less likely to convince skeptics.

One main rational seems to be based on the idea that for any biological problem there is an ideal model organism. This is an old and successful theme that went out of fashion during the early days of molecular genetics due to the lack of resources, particularly mutations, and then later on, assembled genomes. It is coming back, and the authors could clearly outline the history of the concept, state why it is coming back, and how an ENCODE project would help. Another point mentions health, Ag, and environmental needs with absolutely no justification. I imagine that the authors see large numbers of users in industry and research here, but they do not make any statements about how big these industrial communities might be. They should really link the proposal to defined areas of existing interest in things like REACH or Tox21. Various Ag companies spend a lot of money (how much?) doing extensive genotyping and I would guess that the productivity of this enterprise would skyrocket with an appropriate reference annotations for wheat, maize, rapeseed, etc.… These are both important, and each should be more fully developed. Finally, the authors raise the important point that an assembled genome is not very useful without an annotation. Here I think I would discuss some of the universal annotation engines that are under development (e.g. gnomon) and how they make use of biological evidence such as RNA-Seq to build annotations. Again, the neoENCODE project could help those packages work more effectively for minimal amounts of money. The list of questions at the end of the section weaken the argument and don’t contribute to the point the authors are making.

Let detractors write their own critiques.

2) I think it is a mistake to propose a project without some more information on how many species to tackle and maybe this idea should be planted early in the manuscript, rather than waiting until page 5 (the roadmap section itself is very strong). If the idea is to cover a lot of phylogenetic space, I would justify the density of neoENCODE work based on factors such as scientific activity (pubMed search results), quality of existing genomes, number of extant species in the group, economic importance, etc.… The goals need development, and I think that the authors first goal is to identify the important stakeholders and propose a meeting to discuss the issue. This is exactly what happened to launch ENCODE and modENCODE (there were multiple meetings and workshops for about 5 years before the first call for proposal went out at the NIH and I’m sure that similar levels of ground work would be required in the EU or at other granting agencies. If industry is the source, they will require similar due diligence).
3) Natural population data is important, but model organisms also exist in nature, so this particular argument is logical fallacy. That the communities in question have more expertise and interest in natural populations is a much better argument.
4) If communities elect organisms and specific systems, it is not hard to predict what they will vote for. If a reference project gets too tied into the biological interests of the investigators, then the project will fail. It has to be a service oriented infrastructure project. It cannot be just a way for certain labs in “the club” to get funding.
5) I think the lists of types of data that could be generated are too long. Since they really flow directly from ENCODE and ModENCODE, the authors could just have a text box or table. From a practical point of view, I would emphasize the cost/benefit analysis (e.g. tagged transcription factors versus genome-wide footprinting) as learned by the previous projects. Given that the lead time for launching such an initiative could be long, I would also discuss what may be technically possible in five years in addition to what we can do today.
6) I’m not completely clear on what the authors would like readers to do. Are they inviting comments? Published discussion? I still think that the authors should use this manuscript to propose a workshop.

### Reviewer 6

James H. Marden

Department of Biology, Penn State University

I have now read both the initial reviews and what appears to be the revision of this manuscript. I was not among the initial reviewers.

First let me state that I do not know very much about the ENCODE Project and I don’t have time to invest in learning about its particulars at this time. I do know that aspects of the set of Sept 2012 Nature publications and the way the press reported the findings have been sharply criticized. I have read two of those critiques (Dolittle, Graur), both of which focus much attention on estimates of the fraction of non-coding DNA that is functional. The Dolittle critique is measured and reasonable; the Graur critique is more detailed and, points out a number of the pitfalls of BIG SCIENCE (inconsistent definitions, methods, and database procedures). Both critiques point out the glaring absence and need for evolutionary analyses in ENCODE studies. The present manuscript contains a single sentence acknowledging only the existence of these criticisms; it doesn’t grapple with them but rather charges forth to a “Let’s do it!” rallying cry at the end.

There are a number of things about this perspective that don’t sit well with me.

First, I am a typical maverick scientist; I run a small lab and prefer to pose my own questions and design my own approaches. I don’t like to be told what to do and my default state is to distrust and question calls for top-down approaches to scientific endeavors. I’m certainly not alone in having such a nature. Consider for example how Craig Venter found his own way to obtain a human genome sequence while working outside the BIG SCIENCE approach of the Human Genome Project. Venter would not want other people telling him that his work should be organized around a group determined research design, and neither would I.

Second, the viewpoint presented here seems quite unrealistic about the objectives of research on non-model organisms, the best of which utilizes extensive data and analyses (e.g. a rich phylogenetic context; time series and or other dynamics data from nature; spatially explicit population data; responses to ecological variation; well established evolutionary genetics using rigorous statistics and a null hypothesis of neutrality) in order to ask interesting questions about a particular species and to gain some possibly general insights. It is only the worst genomic approaches with non-model species that aim only to obtain a large amount of sequence data without a compelling reason to do so and with little knowledge of what to do with the data. Much of the latter does get published (including in this journal) but that in no way motivates my thinking about the best ways to approach new research.

That said, there is clearly great importance in building and maintaining public databases of sequence data and associated functional annotation, but this paper is not about databases per se. Gaining insights about the genetic mechanisms underlying interesting features of a particular species is often (but not always) a goal of the best genomic research in non-model species. Clearly there will be cases where non-coding regulatory elements are key parts of the answer, but for the vast majority of non-model species research, there will not be a big enough team to undertake an Encode-style approach. Despite such limitations (which the authors acknowledge), they present a perspective that proclaims, metaphorically, that because gold-plated plumbing can now be made and is superior, all subjects in the kingdom should pool their resources and begin installing gold plate on all faucets.

Perhaps most importantly, this perspective does not begin by asking “What is the best way to make new discoveries of importance in non-model species?”. If they asked that question first, and carefully built an argument that the best approach in most cases will be a genome-wide, multi-level informatics approach using a wide array of functional interrogation, then I could respect the paper even if I disagreed with it.

Rather, they seem to assume this as a starting position, but nonetheless conclude that “.... we view these mechanisms as specifically linked to particular biological questions and thus need only be generated on a case-by-case basis.” If the detailed genome-wide molecular mechanisms are not of general interest, but rather there is a subset of interesting mechanisms linked to particular biological questions (a position with which I agree wholeheartedly!), then what is the best scientific approach? Would the best targeted approach look like ENCODE (i.e. interrogate the entire genome for all signals of content, structure and function)? The much better paper would ask and answer 1) what are the positive and negative lessons that non-model species biology can learn from ENCODE, and 2) can, and if so how, do we best apply such approaches in small teams with limited resources interrogating specific biological questions in non-model species?

Major compulsory revisions:

1. Be more concrete early in the paper about what the ENCODE project produced using methods that are transportable to non-model species research at particular levels of scale (e.g. 10 labs, 5 labs, 1 lab).
2. Devote at least a paragraph to the critiques of Dolittle and Gruar. These are important, substantive, and need to be discussed for your perspective to have broad credibility.
3. Early on in the paper, discuss what you think is the best scientific approach for using ENCODE inspired methods on species with restricted biological and financial resources. Provide an example. For instance, perhaps you know of a species in which a trait of interest maps to a particular chromosomal region. Can that region be examined using ENCODE-like approaches, and if so, what are the likely benefits? Do some teaching here because non-model types don’t yet know much about ENCODE and what it has produced.
4. Strive for realism throughout. Be pragmatic. How can we take the first small steps and make incremental progress?
5. Have a native English speaker go over the writing. There are many places where I am unable to determine the meaning.

### Reviewer 7

Jeffrey L. Boore

University of California Berkeley

This is a short opinion piece meant to increase awareness of the importance of ENCODE-like projects for non-model organisms. I am already convinced of the importance of this, myself, so it was mostly just reinforcing my own views, but there are others in the community in greater need of convincing. I recommend acceptance pending a few fairly minor issues.

Major Compulsory Revisions

NONE.

Minor Essential Revisions

Page 1, paragraph 2: “nocturnalcontrary” should read “nocturnal, contrary”

Page 2, line 1, is missing a comma after “transcriptomic” and, if you follow the Oxford method, after “proteomic.”

Page 2, line 4: “trend does not seem to stop any time soon” should read “trend does not seem likely to stop any time soon”

“Biomathematics” is inconsistently hyphenated.

Page 7, lines 2 and 7: “Histones” is inappropriately capitalized.

Page 7, line 13: “through” is misspelled. Add a comma after “. . . a genome)”

Page 7, line 16: Add a comma after “telomeres”.

At three points in the ms., the word “tribune” is used inappropriately.

In the Authors’ Contributions, they state, “DT was at the initiative of this brainstorming” when I think what is meant is “DT initiated this brainstorming”. In addition, I view the word “brainstorming” here as inappropriately colloquial and not really very accurate.

Discretionary Revisions

Page 1, paragraph 2: It seems odd to point out shortcomings for specific subsets of these model organisms that apply to all or most of them just as well. I suggest rewriting this to state the aspects of life that are not addressed by studies of these model organisms and explain why this is important to do so. I question whether “Biomathematics” is a very accurate description of what the authors intend.

I question, too, whether “neoENCODE” is a very accurate description of that the authors intend. Are these organisms really in a category of “newness” relative to the organisms targeted by ENCODE and modENCODE?.

The authors state, “working with non-model organisms means there is no access to high throughput functional assays.” This seems overstated to me, both because there are some high throughput functional assays that are available for some non-model organisms and because of the implication that such assays are readily available for model organisms, which is not always the case. An additional point that could be emphasized is the problem of “percolation of errors” stemming from the use of one genome to annotate the next, on and on, like a game of “telephone.” There may be great value in regularly revisiting assignments of paralogy and orthology as greater numbers of genomes and greater accuracy of gene modeling become available.
